# Radiographic Assessment of Tibiofibular Syndesmosis Injury with Different Durations and Types of Fixation

**DOI:** 10.3390/jcm11216331

**Published:** 2022-10-27

**Authors:** Krzysztof Klepacki, Igor Kowal, Grzegorz Konieczny, Łukasz Tomczyk, Grzegorz Miękisiak, Piotr Morasiewicz

**Affiliations:** 1 Orthopedic Surgery Department, Provincial Specialist Hospital in Legnica, Iwaszkiewicza 5, 59-220 Legnica, Poland; 2 Faculty of Health Sciences and Physical Education, The Witelon State University of Applied Sciences in Legnica, 59-220 Legnica, Poland; 3 Department of Management of Food Quality and Safety, Poznan University of Life Sciences, 60-637 Poznań, Poland; 4 Institute of Medical Sciences, University of Opole, ul. Oleska 48, 45-052 Opole, Poland; 5 Department of Orthopaedic and Trauma Surgery, Institute of Medical Sciences, University of Opole, al. Witosa 26, 45-401 Opole, Poland

**Keywords:** radiographic, tibiofibular syndesmosis injury, duration, types of fixation

## Abstract

Introduction: There is no consensus among orthopedic surgeons on the number of cortical layers (tricortical or quadricortical fixation) involved or the duration of syndesmotic fixation after a tibiofibular syndesmosis (TFSD)-injury treatment. The purpose of this study was to assess radiographic parameters following the treatment of TFSD injuries, with various time-windows of syndesmotic screw removal and numbers of cortical layers involved. Materials and Methods: Fifty-five patients, aged from 25 to 75 years, were included in the study. The follow-up period ranged from 2 years to 4 years and 2 months. The patients were subdivided into groups based on the duration of the syndesmotic fixation (8–15 weeks—19 patients or 16–22 weeks—36 patients) and the number of cortices involved (tricortical—17 patients or quadricortical fixation—38 patients). Results: The quadricortical fixation group showed a significant development of ankle joint arthritis and subtalar joint arthritis at the final follow-up. The mean medial clear space was 2.84 mm in the tricortical fixation group and 3.5 mm in the quadricortical fixation group (*p* = 0.005). Both groups, with different screw removal times showed significant development of posttraumatic arthritis. A comparison of the two groups (with different time-windows of the screw removal) revealed a significant difference only in terms of the postoperative tibiofibular (TF) overlap and the observed rates of talonavicular arthritis at the final follow-up. Discussion: We found that the duration of the screw fixation had no effect on most of the evaluated radiographic parameters. Only the postoperative TF overlap was lower in the 8–15-week fixation group, and the proportion of patients with talonavicular joint arthritis at the final follow-up was higher in the 16–22-week fixation group. In addition, the number of cortices involved in the screw fixation had no effect on the radiographic outcomes in our patients, apart from the differences in one parameter—the medial clear space—at the final follow-up. Conclusion: We achieved similar radiographic results irrespective of the duration of the screw fixation and the number of cortices involved. All study subgroups showed the development of adjacent-joint arthritis following treatment. Considering the results of our study, the economic and medical aspects of treatment, and the possibility of a faster recovery, the most optimal solution seems to be the use of a tricortical fixation, with the screws being removed after 8–15 weeks.

## 1. Introduction

The ankle joint is one of the most common locations of lower limb injuries. Tibiofibular syndesmosis (TFSD) injuries constitute an estimated 5–10% of all ankle injuries [1,2,3,4,5,6,7,8], with the proportion even higher (5–23%) when a TFSD injury is associated with a fracture involving the ankle joint [1,4,5,6,7,8,9,10].

TFSD injuries disrupt the anatomical structure and biomechanics of the ankle joint [1,2,3,5,6,7,8,9,10,11,12,13,14]. The ankle becomes unstable, which leads to an impaired joint function, pain, a limited range of motion, and osteoarthritis [1,2,3,5,6,7,8,9,10,11,12,13,14].

The treatment of TFSD injuries requires ankle joint reconstruction to restore normal biomechanical conditions, reduce pain, improve the functional parameters, and lower the risk of subsequent osteoarthritis [1,2,3,5,6,7,8,9,10,11,12,13,14,15,16,17].

There are many TFSD fixation techniques, with specialists’ opinions being divided as to the optimal one [1,2,3,4,5,6,7,8,9,10,11,13,15]. There is no consensus on the thickness (diameter) or number of screws used to fix a TFSD or the number of cortical layers involved in the fixation [1,3,4,5,6,7,8,9,10,11,13,14,15,16,17].

Some orthopedic surgeons prefer tricortical syndesmosis fixation, arguing that this allows for weight bearing sooner after the surgery, makes for a more dynamic fixation, eliminates the need to remove the screws, and reduces the risk of screw breakage [4,5,6,9,10,11,14]. Other orthopedic surgeons advocate for a quadricortical syndesmosis fixation as the method of choice, due to a greater stability and better anatomical reconstruction of the ankle joint [6,8,9,15].

Moreover, there is no consensus on how long the screw should remain in place [1,3,4,9,11,14,15,16]. Some orthopedic surgeons remove the screw after 8 weeks [3,4,11,14,15], some after 12 weeks [14], and some after 16–20 weeks [16]. There are also those who believe that the screw should not be removed from the syndesmosis, as the screw removal necessitates another surgical procedure, which increases the risk of complications, particularly infections, and generates additional costs [1,9].

Many reports emphasize the fact that TFSD injuries are associated with subsequent osteoarthritis [3,5,6,7,8,9,11,13]; however, there have been no studies to assess the presence of adjacent-joint arthritis following a TFSD-injury treatment.

Evaluating TFSD-injury treatment outcomes should involve comprehensive radiographic and biomechanical as well as clinical assessments [2,4,5,6,8,9,11,12,13,15,16,17]. Previous studies focused on the surgical techniques [5], clinical assessments [3,4,5,7,9,11,14], and functional assessments [3,4,14] used in managing TFSD injuries. Few studies, and only to a limited extent, have analyzed radiographic parameters following a TFSD-injury treatment [5,8,9,11,16].

We hypothesized that the period of time to the screw removal and the number of cortices involved in the fixation would affect radiographic outcomes in TFSD-injury patients.

The purpose of this study was to assess the radiographic parameters following the treatment of TFSD injuries, with various time-windows of the syndesmotic screw removal and the numbers of cortical layers involved.

## 2. Material and Methods

During the period from 2017 to 2019, there were 151 patients with TFSD injuries treated at our department. The inclusion criteria were: a TFSD injury treated in our department; an unstable Webber type B or C ankle fracture; trauma radiographs showing evidence of tibiofibular (TF) ligament damage, such as a TF overlap of <10 mm, a TF diastasis of >5 mm, and a medial clear space of >4 mm [2,4,5,6,8,9,12]; a follow-up period of at least 2 years after treatment completion; complete clinical and radiographic records; no other lower limb pathologies; no neurological conditions; and an informed consent.

The exclusion criteria were: a TFSD injury treated somewhere else other than at our department; no unstable Webber type B or C ankle fractures with a concomitant TFSD injury; a follow-up period of less than 2 years after treatment completion; incomplete clinical and radiographic records; concomitant lower limb pathologies; neurological conditions; no informed consent, and a bilateral ankle injury. The application of the inclusion and exclusion criteria resulted in 96 patients being disqualified from taking part in the study.

The study was conducted according to the guidelines of the Declaration of Helsinki and approved by Bioethics Committee at the Lower Silesian Medical Chamber (protocol code 1/PNDR/2021; 13.01.2021). All study subjects were informed of the voluntary nature of their participation and the option of withdrawing from the study at any time, and all of them gave their written consent.

The use of the inclusion and exclusion criteria resulted in 55 patients (37 females and 18 males), aged from 25 to 75 years, being ultimately included in the study. The patients’ heights ranged from 165 to 190 cm, body weights—from 50 to 110 kg, and body mass indexes (BMI)—rom 17 to 40. The follow-up period from treatment completion to the final follow-up assessment ranged from 2 years to 4 years and 2 months.

The patients were subdivided into groups based on the duration of the syndesmotic fixation (8–15 weeks or 16–22 weeks) and the number of cortices involved (tricortical or quadricortical fixation).

The subgroup with an 8–15-week fixation comprised 19 patients, and the subgroup with a 16–22-week fixation comprised 36 patients. Seventeen patients underwent a tricortical fixation, whereas 38 patients underwent a quadricortical fixation for their TFSD injury.

The unstable Webber type B or C fractures associated with a TFSD injury were treated with an open reduction and internal fixation. The surgeries were performed with the use of C-arm fluoroscopy. The fracture was exposed via a lateral incision over the fibula. The fracture was reduced and temporarily fixed with a Kirschner wire (K-wire). Once the bone fragments were adequately repositioned, the fibular fracture was fixed with the use of an anatomic plate. The subsequent stage involved the use of a lag screw to reposition and fix the injured TFSD. Based on the bone quality, the fracture type, the TFSD-injury type, and the individual preferences, the operator decided to use a tricortical or quadricortical fixation of the injured TFSD with a lag screw of 3.5 mm in diameter.

One of a group of three orthopedic surgeons performed each patient’s operation. An adequate syndesmosis reduction in each patient was confirmed via C-arm fluoroscopy and postsurgical radiographs. The correctness of the bone fragment repositioning and fixation was assessed based on the ankle congruence, the TF overlap, the TF diastasis, and the medial clear space.

During the postoperative period, patients walked with the help of two elbow crutches, without bearing full weight on the operated limb and were instructed to maintain ankle mobility. At week 6 after the procedure, bone union was assessed radiographically, and the patients underwent rehabilitation and syndesmotic screw removal surgery. Based on the radiographic findings, the patients were allowed to gradually increase weight bearing on the operated limb until full weight bearing occurred. The fracture healing was defined as the union of bone fragments on the radiographs (trabecular transition or the connection of bone cortical layers), no pain, and pathological mobility at the fracture site when force was applied or during walking with full weight bearing. Moreover, depending on the current condition of the operated ankle, operating room availability, and the patients’ personal schedules, the syndesmotic screw removal procedure was scheduled and performed within one of the two time-windows following the initial surgery. The rehabilitation protocol was identical for all patients.

The following parameters were assessed in this study: TF diastasis, TF overlap, the medial clear space, adjacent joint arthritis, screw loosening, screw breakage, and tibiofibular syndesmosis ossification.

A normal TF distance was considered to be <5 mm [2,4,5,6,8,9,12]. A normal TF overlap was considered to be >10 mm [2,4,5,6,8,9,12]. The medial clear space was considered to be normal at <4 mm [2,4,5,6,8,9,12].

The long-term follow-up included the assessment of the parameters indicating the maintained ankle congruence (TF diastasis, TF overlap, and medial clear space) and the presence of adjacent-joint arthritis. The tibiotalar, subtalar, talonavicular, and calcaneocuboid joints were evaluated for evidence of arthritis in comparison with that in earlier radiographs [18]. Arthritis was defined as the occurrence of degenerative changes on the AP and lateral radiographs according to the five-grade Kellgren–Lawrence scale [19]. Grades ≥ 2 were considered indicative of degenerative changes. The following complications were considered: screw loosening, screw breakage, and tibiofibular syndesmosis ossification. Radiographic evaluation was conducted based on the weight-bearing AP and lateral views of the ankle joint and foot.

Data were statistically analyzed using Statistica 13.1. The Shapiro–Wilk test was used to check for normality of distribution. Student’s *t*-test was used to compare quantitative variables. For qualitative variables, Pearson’s chi-square test was used. The level of statistical significance was set at *p* < 0.05.

## 3. Results

The study groups showed no significant differences in terms of the patients’ age or follow-up duration (Table 1 and Table 2).

On the final follow-up assessment, both the tricortical and quadricortical TFSD fixation subgroups showed a significantly decreased TF overlap, beyond the established normal values (Table 3).

The TF overlap in the tricortical fixation group was 10.19 mm postoperatively and 7.01 mm at the final follow-up (*p* = 0.003); in the quadricortical fixation group it was 10.11 mm postoperatively and 8.26 mm at the final follow-up (*p* = 0.0019). The quadricortical fixation group also showed a significant increase in TF diastasis from 2.59 mm postoperatively to 4.09 mm at final follow-up (*p* = 0.0002). Both groups showed a significant reduction of the medial clear space from the postoperative to the respective final follow-up values; from 3.72 mm to 2.84 mm in the tricortical fixation group and from 4.11 mm to 3.5 mm in the quadricortical fixation group (Table 3). However, the medial clear space values did not exceed the normal limits. The quadricortical fixation group showed a significant development of ankle joint arthritis, which was observed in 2.63% of patients postoperatively and in 63.15% of patients at the final follow-up (*p* = 0.0001) (Table 3). This subgroup also showed increased rates of subtalar joint arthritis, from 10.52% postoperatively to 47.36% at the final follow-up (*p* = 0.0017) (Table 3. No increase in arthritis rates was observed in the tricortical fixation group at the final follow-up.

**Figure 1:**
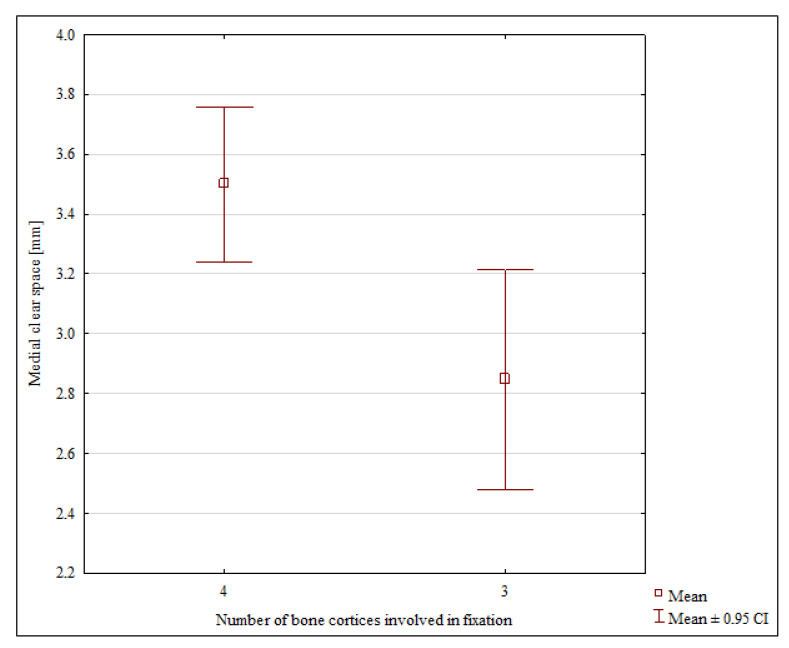
Medial clear space values in the tricortical and quadricortical fixation groups.

A comparison of the tricortical and quadricortical fixation groups revealed no significant differences in the values of most of the individual radiographic parameter values (Table 1). The only parameter that did show a significant difference between the groups at the final follow-up was the medial clear space. The mean medial clear space was 2.84 mm in the tricortical fixation group and 3.5 mm in the quadricortical fixation group (*p* = 0.005) (Table 1, Figure 1).

There was a total of eight complications (in 21% of patients) in the quadricortical fixation group; these were three loosened screws, one screw breakage, and four cases of tibiofibular syndesmosis ossification. There was a total of three complications (in 17.6% of patients) in the tricortical fixation group; these were two broken screws and one loosened screw. The two groups showed no significant differences in terms of the complication rates (Table 1).

The analysis of the study population stratified by the duration of the syndesmotic fixation showed that the two subgroups differed significantly in the TF overlap and the medial clear space between the postoperative and the final follow-up assessments (Table 2). In both the evaluated subgroups, the medial clear space values were within the established normal limits. The 16–22-week fixation group also showed an increase in the TF diastasis values from 2.63 mm postoperatively to 3.95 mm at the final follow-up (*p* = 0.0012) (Table 4); this parameter was within normal limits at the final follow-up.

Both groups showed significant development of posttraumatic arthritis (Table 4). In the 8–15-week fixation group, the arthritis rates increased in the ankle joints (an increase from 5.26% postoperatively to 57.89% at the final follow-up) (*p* = 0.0021) and the talonavicular joints (an increase from 0% postoperatively to 31.57% at the final follow-up) (*p* = 0.0076). In the 16–22-week fixation group, a significant increase in arthritis was observed in the ankle joints (an increase from 5.55% postoperatively to 55.55% at the final follow-up) (*p* = 0.0006) and in the subtalar joints (an increase from 11.11% postoperatively to 44.44% at the final follow-up) (*p* = 0.0015).

**Figure 2:**
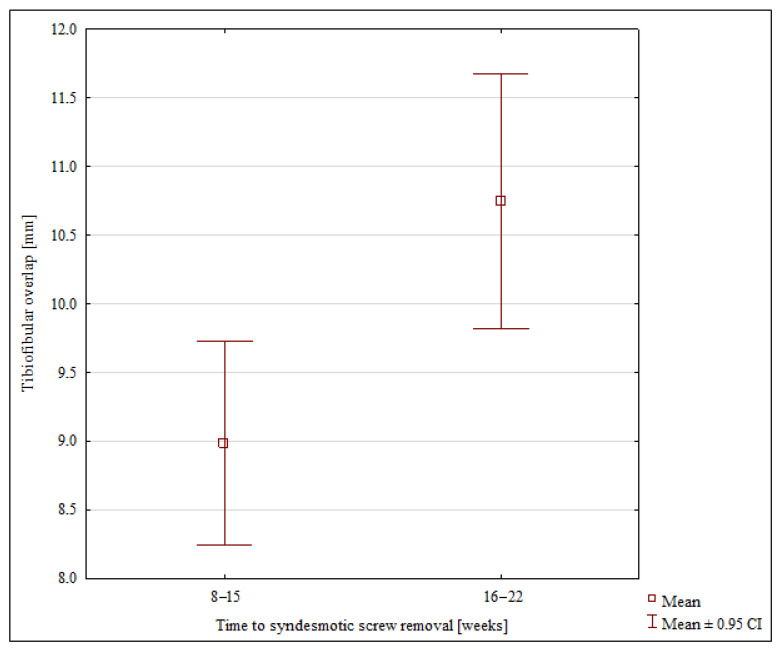
Postoperative tibiofibular overlap values in the 8–15 and 16–22-week fixation groups.

**Figure 3:**
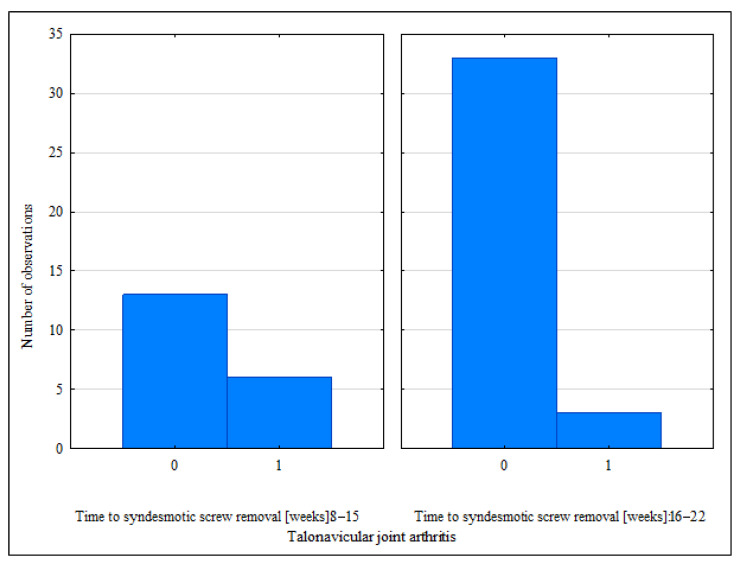
Talonavicular joint arthritis at the final follow-up in the 8–15 and 16–22-week groups.

A comparison of the two groups with the different time-windows of screw removal revealed a significant difference only in terms of the postoperative TF overlap and the observed rates of talonavicular arthritis at the final follow-up (Table 2) (Figure 2 and Figure 3).

Patients from the 16–22-week fixation group experienced three cases of screw breakage, four cases of screw loosening, and one case of tibiofibular syndesmosis ossification. Patients from the 8–15-week fixation group experienced three cases of tibiofibular syndesmosis ossification. The differences in complication rates between these groups were not statistically significant (Table 2).

## 4. Discussion

Our study assessed the effects of the time of screw removal and the number of cortices involved in fixation on the radiographic outcomes following a TFSD injury. These aspects of the TFSD-injury treatment have not been evaluated extensively in earlier studies. We found that the duration of screw fixation had no effect on most of the evaluated radiographic parameters. Only the postoperative TF overlap was lower in the 8–15-week fixation group, and the proportion of patients with talonavicular joint arthritis at the final follow-up was higher in the 16–22-week fixation group. In addition, the number of cortices involved in screw fixation had no effect on the radiographic outcomes in our patients, apart from the differences in one parameter—the medial clear space—at the final follow-up. These results do not support our study hypothesis.

TFSD injuries disrupt the anatomical structure and biomechanics of the ankle joint and result in uneven weight distribution within the joint [1,2,3,5,6,7,8,9,10,12,13,14,15,16,17]. Prompt and effective treatment of TFSD injuries is essential for restoring ankle joint stability, improving joint function, reducing pain, improving joint mobility, and lowering the risk of arthritic complications [1,2,3,5,7,8,9,10,11,12,13,14].

Orthopedic surgeons vary in terms of their preferred TFSD fixation techniques [1,4,8,10]. Some choose tricortical syndesmosis fixation [10,11], some prefer quadricortical syndesmosis fixation [1,8,9], whereas some report similar outcomes irrespective of the fixation technique [1,4,9,11,14].

The authors advocating for tricortical syndesmosis fixation argue that it allows for faster postoperative weight-bearing, makes for a more dynamic fixation, reduces the range of motion limitations in the ankle joint, does not require screw removal, and lowers the risk of screw breakage [1,4,5,9,10,11,14]. Since this technique does not require the screw to be removed, there is no need for a reoperation, which would increase the risk of complications and increase treatment costs and the number of days the patient is unable to work [5,9,10]. Nonetheless, some orthopedic surgeons use quadricortical syndesmosis fixation as the method of choice, due to a better ankle joint stability and the possibility of better anatomical reconstruction of the joint, as well as an easier removal of the screw in case of its breakage, as both ends of the screw protrude from the tibia [1,8,9,15].

There is also no consensus on the optimal period of time that the screw should remain in the syndesmosis [1,3,4,9,11,15,16]. Some surgeons remove the screw after 8 weeks [3,4,11,14,15], some after 12 weeks [14], and some after 16–20 weeks [16]. There are also those who believe that the screw should not be removed from the syndesmosis, as this requires an additional surgery, which increases the risk of complications, particularly infections, and generates additional costs [1,5,9].

Many authors emphasize that TFSD injury leads to the development of osteoarthritis [3,5,8,9,11,13]; however, there are no studies assessing the presence of adjacent-joint arthritis following a TFSD-injury treatment.

In assessing the treatment outcomes after a TFSD injury, it is important to comprehensively evaluate the radiographic and biomechanical as well as the clinical parameters [2,4,5,8,9,11,12,13,16,17]. The aspect of radiographic outcomes following a TFSD-injury treatment has not been extensively investigated and is not fully known [5,8,9,11,16]. A systematic review by Zhang showed a malreduction in 12.6% of patients after a TFSD-injury fixation with a screw, an implant failure in 30.9% of patients, and other complications in 16.4% of patients [5]. Stuart reported screw breakages in 10% of patients and screw loosening in 12% of patients following a syndesmosis fixation [8]. In the group of patients evaluated by Kaftandziev et al. 16% of patients experienced screw breakages [9]. Hoiness reported screw breakages in 14.7% of patients and screw loosening in 5.8% of patients following a tricortical syndesmosis fixation [14]. Walker et al. observed screw breakages in 25% of patients, and screw loosening in 22.2% of patients [16]. Our study showed no effect of the duration of the screw fixation or the number of bone cortices involved on the complication rates. In our study population we observed slightly lower complication rates than those reported in the literature [5,8,9,14,16].

Schepers et al. observed no differences in the American Orthopedic Food and Ankle Society (AOFAS) score, the Olerung and Molander Ankle Score (OMAS), or the visual analog scale (VAS) score, irrespective of the period of time after which the syndesmotic screw was removed [4]. Our study findings indicated that the duration of the screw fixation had no effect on most of the evaluated radiographic parameters. It was only the postoperative TF overlap that was below the lower level of normal in the 8–15-week fixation group, and the proportion of patients with talonavicular joint arthritis at the final follow-up was significantly higher in the 16–22-week fixation group. A comparison of the parameters assessed postoperatively and at the final follow-up in the two groups with a different duration of screw fixation revealed the development of arthritis and a worsened TF overlap.

Schepers et al. reported no differences in the AOFAS, OMAS, or VAS scores in the evaluated patients with a different number of bone cortices involved in the screw fixation [4]. Stuart observed screw breakages in 10% of patients after a tricortical syndesmosis fixation and none in patients after a quadricortical syndesmosis fixation [8]. Wikroy et al. observed no differences in the OMAS, Orthopedic Trauma Association (OTA) score, or the number of arthritic joints following treatment with either a tricortical or a quadricortical fixation [11]. The patient population evaluated by Hoiness and Stromsoe showed no differences in terms of pain, dorsiflexion, or functional scores between the tricortical and quadricortical syndesmosis fixation groups [14]. Markolf et al. reported no effect of the number of screws or the engaged tibial cortices on the mechanical stability of the distal fibula [15]. This is consistent with the results of our study, where the number of bone cortices involved in the screw fixation had no effect on the radiographic outcomes in our patients, with the exception of one parameter, the medial clear space, which showed differences between the study groups at the final follow-up (nonetheless, this parameter was within normal limits in both groups). The TF-overlap values measured in both groups at the final follow-up were beyond the limits adopted as normal and were lower than the TF-overlap values that were measured postoperatively. The two subgroups that were differing in the number of engaged cortical layers showed the development of adjacent-joint arthritis at the final follow-up.

The presence of adjacent-joint arthritis at the final follow-up may indicate the biomechanical disturbance of the ankle and the foot having persisted after treatment, irrespective of the duration of the screw fixation or the number of cortices involved. Moreover, foot and ankle arthrosis following a TFSD injury may lower the TF-overlap values below the lower limit of normal for a long time after treatment.

The limitations of our study include its retrospective character; however, due to the traumatic nature of the TFSD injuries, it is impossible to assess the patients prior to the treatment. Other studies assessing TFSD injuries are also retrospective [3,7,16]. Another limitation of our study was the lack of randomization into groups, which makes it very difficult to draw conclusions. The strengths of our study are the large sample size, the operators being from a group of three orthopedic surgeons, and the same rehabilitation protocol for all the patients.

The results of this study suggest that a tricortical fixation may be more beneficial for several reasons. Firstly, due to the similar radiographic outcomes irrespective of the number of bone cortices involved; secondly, due to the necessity of removing the syndesmotic screw in the case of a quadricortical fixation (which increases treatment costs and the risk of postoperative complications); thirdly, the screw removal increases the number of days the patient is unable to work.

Since the radiographic outcomes were similar, we prefer an earlier screw removal, as this helps shorten the treatment and rehabilitation periods and accelerates the return to work and the regaining of foot functionality.

## 5. Conclusions

We achieved similar radiographic results irrespective of the duration of the screw fixation and the number of cortices involved.

All study subgroups showed the development of adjacent-joint arthritis following treatment.

The study groups did not differ in terms of complication rates.

Considering the results of our study, the economic and medical aspects of treatment, and the possibility of a faster recovery, the most optimal solution seems to be the use of a tricortical fixation, with the screw removal after 8–15 weeks.

## Figures and Tables

**Table 1:** Detailed results after treatment with 3 and 4 stabilized cortices.

Analyzed Variable (Mean ± Standard Deviation)	3 Corticles Group n = 17	4 Corticles Group n = 38	*p* Value
Age of patients [years]	54.75 ± 14.57	48.65 ± 15.04	0.1664 *
Observation period [months]	40.05 ± 14.81	39.22 ± 11.31	0.8203 *
TF overlap after surgery [mm]	10.19 ± 2.01	10.11 ± 2.74	0.9107 *
TF diasthasis after surgery [mm]	3.55 ± 1.98	2.59 ± 1.56	0.0573 *
Medial clear space after surgery [mm]	3.72 ± 0.812	4.11 ± 0.94	0.1548 *
Ankle joint arthritis after surgery [%]	11.76	2.63	0.1681 **
Subtalar joint arthritis after surgery [%]	23.52	10.52	0.2062 **
Talonavicular joint arthritis after surgery [%]	5.88	5.26	0.9255 **
Calcaneocuboid joint arthritis after surgery [%]	5.88	7.89	0.7905 **
TF overlap Long term evaluation [mm]	7.01 ± 3.7	8.26 ± 2.24	0.1267 *
TF diasthasis Long term evaluation [mm]	4.04 ± 1.69	4.09 ± 1.8	0.9257 *
Medial clear space Long term evaluation [mm]	2.84 ± 0.71	3.5 ± 0.78	0.005 *
Ankle joint arthritis Long term evaluation [%]	41.17	63.15	0.381 **
Subtalar joint arthritis Long term evaluation [%]	41.17	47.36	0.7137 **
Talonavicular joint arthritis Long term evaluation [%]	29.41	10.52	0.08 **
Calcaneocuboid joint arthritis Long term evaluation [%]	17.64	7.89	0.2836 **
Screw loosening	1	3	0.7905 **
Screw breakage	2	1	0.1681 **
Tibiofibular syndesmosis desis	0	4	0.1167 **
All complications	3	8	0.1432 **

*—Student’s *t* test. **—Pearson’s chi-squared test.

**Table 2:** Detailed results after screw hold time 8–15 weeks and 16–22 weeks.

Analyzed Variable (Mean ± Standard Deviation)	8–15 Weeks Group n = 19	16–22 Weeks Group n = 36	*p* Value
Age of patients [years]	50.11 ± 13.32	50.76 ± 16.04	0.88 *
Observation period [months]	42.46 ± 13.71	37.91 ± 11.47	0.197 *
TF overlap after surgery [mm]	8.98 ± 1.54	10.74 ± 2.73	0.012 *
TF diasthasis after surgery [mm]	3.38 ± 1.83	2.63 ± 1.65	0.1258 *
Medial clear space after surgery [mm]	4.03 ± 0.97	3.97 ± 0.9	0.8321 *
Ankle joint arthritis after surgery [%]	5.26	5.55	0.9337
Subtalar joint arthritis after surgery [%]	21.05	11.11	0.32
Talonavicular joint arthritis after surgery [%]	0	8.33	0.1956
Calcaneocuboid joint arthritis after surgery [%]	5.26	8.33	0.6767
TF overlap Long term evaluation [mm]	7.2 ± 2.47	8.23 ± 2.92	0.1993 *
TF diasthasis Long term evaluation [mm]	4.31 ± 1.92	3.95 ± 1.67	0.4729 *
Medial clear space Long term evaluation [mm]	3.22 ± 0.85	3.33 ± 0.8	0.64 *
Ankle joint arthritis Long term evaluation [%]	57.89	55.55	0.4823
Subtalar joint arthritis Long term evaluation [%]	47.36	44.44	0.1366
Talonavicular joint arthritis Long term evaluation [%]	31.57	8.33	0.0267
Calcaneocuboid joint arthritis Long term evaluation [%]	15.78	8.33	0.3989
Screw loosening	0	4	0.1313
Screw breakage	0	3	0.1965
Tibiofibular syndesmosis desis	3	1	0.2093
All complications	3	8	0.2141

*—Student’s *t* test.

**Table 3:** Detailed radiological assessment of patients after surgery and in last control visit in the 3 corticles group and 4 corticles group.

Analyzed Variable [MEAN]	after Surgery	Long Term Evaluation	*p* Value
**3 corticles group**			
Ankle joint arthritis [%]	11.76	41.17	0.1353 **
Subtalar joint arthritis [%]	23.52	41.17	0.4083 **
Talonavicular joint arthritis [%]	5.88	29.41	0.719 **
Calcaneocuboid joint arthritis [%]	5.88	17.64	0.287 **
TF overlap after surgery [mm]	10.19 ± 2.01	7.01 ± 3.7	0.003 *
TF diasthasis after surgery [mm]	3.55 ± 1.98	4.04 ± 1.69	0.4455 *
Medial clear space [mm]	3.72 ± 0.81	2.84 ± 0.71	0.002 *
**4 corticles group**			
Ankle joint arthritis [%]	2.63	63.15	0.0001 **
Subtalar joint arthritis [%]	10.52	47.36	0.0017 **
talonavicular joint arthritis [%]	5.26	10.52	0.3949 **
Calcaneocuboid joint arthritis [%]	7.89	7.89	0.7841 **
TF overlap after surgery [mm]	10.11 ± 2.74	8.26 ± 2.24	0.0019 *
TF diasthasis after surgery [mm]	2.59 ± 1.56	4.09 ± 1.8	0.0002 *
Medial clear space [mm]	4.11 ± 0.94	3.5 ± 0.78	0.003 *

*—Student’s *t* test. **—Pearson’s chi-squared test.

**Table 4:** Detailed radiological assessment of patients after surgery and in last control visit in the 8–15 weeks group and 16–22 weeks group.

Analyzed Variable (Mean ± Standard Deviation)	after Surgery	Long Term Evaluation	*p* Value
**8–15 weeks group n = 19**			
Ankle joint arthritis [%]	5.26	57.89	0.0021 **
Subtalar joint arthritis [%]	44702,00	47.36	0.1482 **
Talonavicular joint arthritis [%]	0	31.57	0.0076 **
Calcaneocuboid joint arthritis [%]	5.26	15.78	0.2904 **
TF overlap after surgery [mm]	8.98 ± 1.54	7.2 ± 2.47	0.0116 *
TF diasthasis after surgery [mm]	3.38 ± 1.83	4.31 ± 1.92	0.1374 *
Medial clear space [mm]	4.03 ± 0.97	3.22 ± 0.85	0.0101 *
**16–22 weeks group n = 36**			
Ankle joint arthritis [%]	5.55	55.55	0.0006 **
Subtalar joint arthritis [%]	44876,00	44.44	0.0015 **
Talonavicular joint arthritis [%]	8.33	8.33	0.9121 **
Calcaneocuboid joint arthritis [%]	8.33	8.33	0.8541 **
TF overlap after surgery [mm]	10.74 ± 2.73	8.23 ± 2.92	0.0003 *
TF diasthasis after surgery [mm]	2.63 ± 1.65	3.95 ± 1.67	0.0012 *
Medial clear space [mm]	3.97 ± 0.9	3.33 ± 0.8	0.0023 *

*—Student’s *t* test. **—Pearson’s chi-squared test.

## Data Availability

The datasets used and/or analyzed during the current study are available from the corresponding author on reasonable request. The data are not publicly available due to privacy.
